# Smartphone-Based LiDAR Application for Easy and Accurate Wound Size Measurement

**DOI:** 10.3390/jcm12186042

**Published:** 2023-09-19

**Authors:** Bokeun Song, Jeonghee Kim, Hyeokjae Kwon, Sunje Kim, Sang-Ha Oh, Yooseok Ha, Seung-Han Song

**Affiliations:** 1 Department of Plastic and Reconstructive Surgery, Chungnam National University Hospital, Daejeon 35015, Republic of Korea; bogenlove@gmail.com (B.S.); bogenlove@cnuh.co.kr (J.K.); kwon.hyeokjae@cnuh.co.kr (H.K.); kkk9243@naver.com (S.K.); djplastic4073@gmail.com (S.-H.O.); 2 Department of Medical Science, College of Medicine, Chungnam National University, Daejeon 35015, Republic of Korea; 3 Department of Plastic and Reconstructive Surgery, College of Medicine, Chungnam National University, Daejeon 35015, Republic of Korea

**Keywords:** surgical wound, dimensional measurement accuracy, surgical flap

## Abstract

The accurate assessment of wound size is a critical step in advanced wound care management. This study aims to introduce and validate a Light Detection and Ranging (LiDAR) technique for measuring wound size. Twenty-eight wounds treated from December 2022 to April 2023 at the Chungnam National University Hospital were analyzed. All the wounds were measured using three techniques: conventional ruler methods, the LiDAR technique, and ImageJ analysis. Correlation analysis, linear regression, and Bland–Altman plot analysis were performed to validate the accuracy of the novel method. The measurement results (mean ± standard deviation) obtained using the ruler method, LiDAR technique, and ImageJ analysis were 112.99 ± 110.07 cm^2^, 73.59 ± 72.97 cm^2^, and 74.29 ± 72.15 cm^2^, respectively. The Pearson correlation coefficient was higher for the LiDAR application (0.995) than for the conventional ruler methods (mean difference, −5.0000 cm^2^), as was the degree of agreement (mean difference, 38.6933 cm^2^). Wound size measurement using LiDAR is a simple and reliable method that will enable practitioners to conveniently assess wounds with a flattened and irregular shape with higher accuracy. However, non-flattened wounds cannot be assessed owing to the technical limitations of LiDAR.

## 1. Introduction

The ability to accurately and precisely measure wound size is critical for wound assessment because it determines the overall effectiveness of wound management [1]. However, physicians and nurses typically use the traditional method involving rectangular measurements with a ruler. These measurements are quick but can be inaccurate and unreliable, especially for irregularly shaped wounds. In addition, this ruler-based rectangular measurement tends to overestimate the actual size of the wound, and the reliability of this technique decreases as the wound size increases [2]. Thus, the ruler measurement technique is unreliable, with up to 44% error in measurements [1,2,3]. A relatively accurate method for measuring the wound size involves tracing the wound perimeter on a transparent film and calculating the size of the wound either manually or using computer-assisted methods. Tracing generally ensures a better representation of the wound area than measuring using a ruler; however, it is considerably time-consuming and increases the risk of wound contamination and patient discomfort due to contact with the film [4].

**Figure 1:**
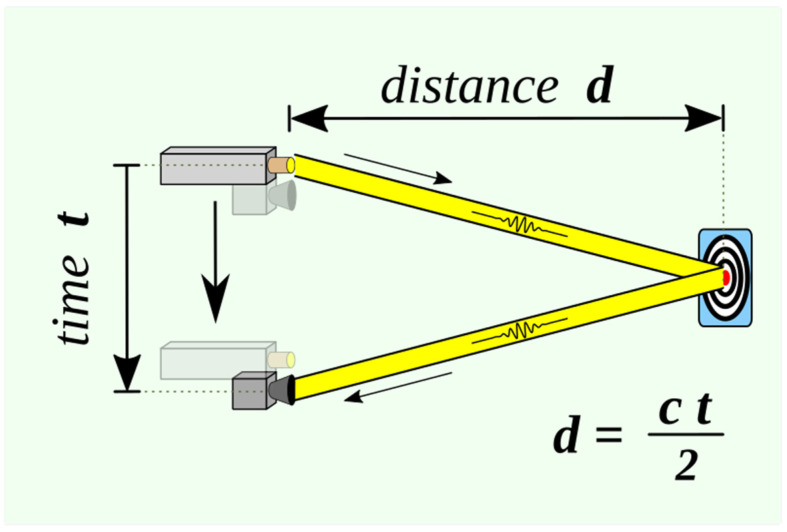
Basic time-of-flight principles used in laser range finding.

Light Detection and Ranging (LiDAR) is a common technique for measuring distances, which functions by timing the return pulse emitted from a laser transmitter to a laser receiver [1]. Lasers with wavelengths of 500–600 nm are typically used in ground-based LiDAR systems, whereas those with wavelengths of 1000–1600 nm are used in airborne LiDAR systems. LiDAR can be used to determine the distance between the detector and an object or surface using the following formula (Figure 1):

$$d=\frac{c*t}{2}$$

where *c* is the speed of light and *d* is the distance between the detector and the object or surface being detected.

The LiDAR technology has been used in aerospace and industrial applications to measure a wide range of areas and heights since the 1960s. With the recent introduction of the LiDAR technology in iPhone cameras, this technology is currently being widely used in interior architecture and building measurements [1,2]. The application of the LiDAR technique in flap size measurement or wound surface area measurement can have advantages; hence, in this study, we use accurate and simple LiDAR-based smartphone applications that do not require a probe for calibration.

In this study, we aim to compare the accuracy of the aforementioned LiDAR-based smartphone applications in measuring wound sizes and compare the results to those obtained using the traditional ruler measurement and ImageJ computational measurement methods.

## 2. Materials and Methods

### 2.1. Study Design and Patient Selection

The study was performed between December 2022 and April 2023 at the Department of Reconstructive and Plastic Surgery of Chungnam National University Hospital. Patients aged over 18 years were eligible for enrolment in this study. Approval to conduct this human-subject-based study was obtained from the Institutional Review Board of Chungnam National University Hospital, and all procedures complied with the ethical rules for human experimentation stated in the 1975 Declaration of Helsinki (CNHU-IRB-2023-06-098).

This prospective study evaluated 45 patients (18 males and 27 females) who underwent reconstructive surgery during the study period. Various patients who underwent surgeries ranging from simple excisional biopsies, local flap for pressure sore reconstruction, abdominoplasty, and free flap surgery to lower leg reconstruction were included.

**Figure 2:**
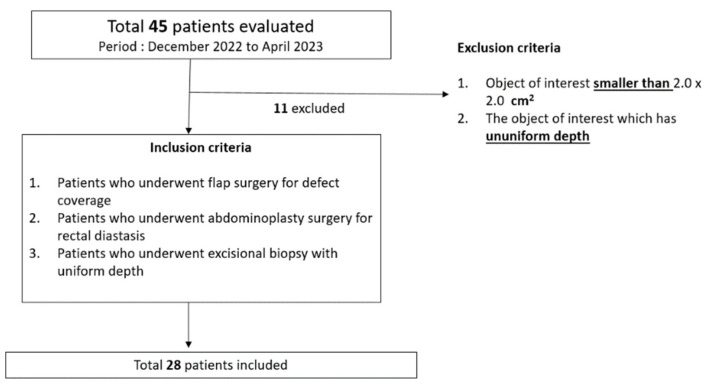
Eligibility criteria (inclusion and exclusion criteria).

The eligibility criteria for this study were as follows (Figure 2):

- Exclusion criteria:
  ○ Object of interest (mass, wound, or flap) smaller than 2.0 × 2.0 cm^2^.
  ○ Object of interest (mass, wound, or flap) with uneven depth that may cause inaccurate recognition by LiDAR-based surface-area scanning.
- Inclusion criteria:
  ○ Patients who underwent flap surgery for defect coverage.
  ○ Patients who underwent abdominoplasty surgery for rectal diastasis.
  ○ Patients who underwent excisional biopsy with uniform depth.

Wounds smaller than 2.0 × 2.0 cm^2^ were not included because of the technical limitations of the LiDAR-based surface-area scanning performed using smartphone devices. Wounds smaller than this size could not be measured because the size of the dot on which the application was segmented was larger than the size of the mass.

As two-dimensional planes are required to obtain surface-area values, free-flap donors and their associated defects were assessed as representative owing to the corresponding topological properties. Hence, only flat wounds or flaps were included in this study.

**Figure 3:**
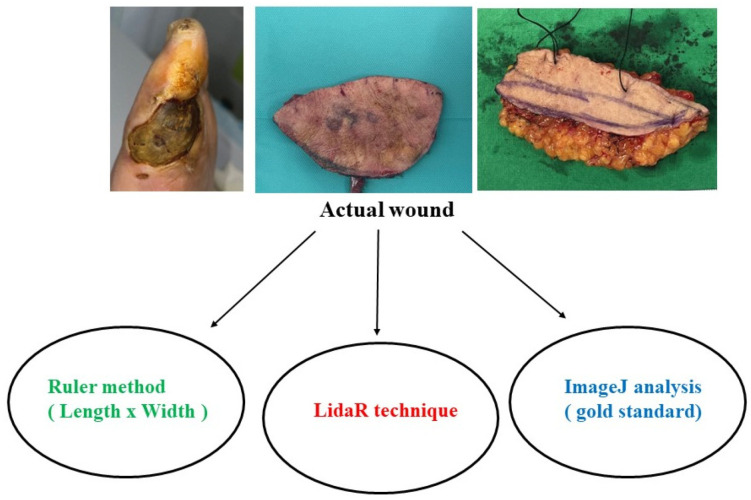
Comparison of the three different measurement methods used in this study.

Each flap and its associated defects were measured using three different methods. The subcutaneous tissue beyond the skin was excluded from the measurement of interest based on the skin portion of the flap and its associated defects. In total, 28 wounds were assessed using three techniques: the conventional ruler method, a LiDAR technique-based smartphone application, and ImageJ analysis (Table 1 and Figure 3).

### 2.2. Measurement Methods

(1) Traditional ruler-based rectangular method

For each of the wounds, the width and length (cm) of the wound were measured using a ruler using the traditional rectangular measurement technique; we assumed the longest length of the area of interest as the major axis and the longest length at right angles as the minor axis and multiplied the two values to calculate the area.

(2) LiDAR technique-based method (AR Ruler App: Tape Measure Cam, GRYMALA Company, Minsk, Belarus)

For each of the 28 flaps and their associated defects, the size of the area was measured using the AR Ruler installed on an iPhone 12 Max Pro (Apple inc., Cupertino, CA, USA). The hardware performance of the devices are described below, and the actual measurement method is demonstrated in a video clip (Supplementary Video). Specific hardware information and a list of smartphones (or devices) compatible with the LiDAR technique are provided below (Table 2).

(3) ImageJ analysis

Photographs of the patients’ wounds were captured using a smartphone camera. The original photographs were then copied to a new folder. The wound area was measured using the following previously described method [4]:

1. ImageJ software(version 1.53t) was opened.
2. File > Open (Alternatively, the picture can be dragged and dropped into the software).
3. A segment was drawn along the ruler using the “straight line” tool. The examiners drew a 1 cm line along the ruler. The software then calculated the distance in the pixels of the segment.
4. Analyze menu > set scale > known distance (we used 1) > unit of length (cm). The software automatically recalculated the number of pixels in cm.
5. The wound outline was created using the “Freehand selections” tool by tracing the wound shape with the computer mouse (desktop computers) or trackpad (laptops).
6. Analyze menu > measure. The area was calculated in cm^2^.
7. A screenshot was taken of the screen along with the measurement window to save in the database.

Each technique was measured by three different physicians to avoid measurement bias.

### 2.3. Statistical Analyses

The mean value of each wound size measurement and the differences between them were analyzed using a paired t-test. The correlation between the two measurements was calculated using Pearson correlation. Linear regression and Bland–Altman plot analyses were performed to statistically examine the agreement between the different measurement methods. The results were considered statistically significant at *p* < 0.05. Data obtained from the study were analyzed using the Statistical Package for the Social Sciences version 26 (IBM Corporation, Armonk, NY, USA).

## 3. Results

### 3.1. Statistical Interpretation

In total, 28 wounds were assessed using three techniques: the conventional ruler method, a LiDAR technique-based smartphone application, and ImageJ analysis.

**Figure 4:**
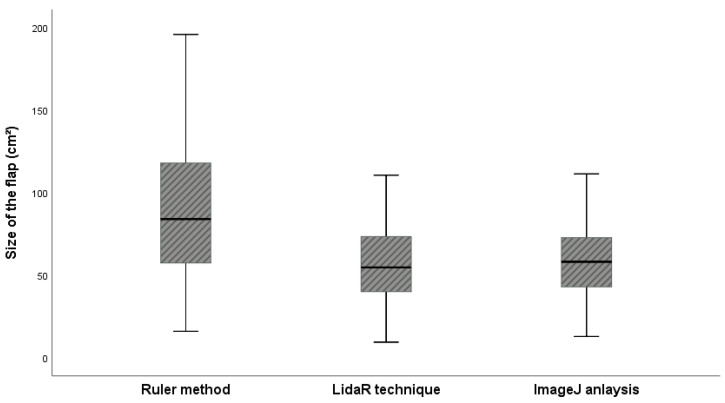
Box plot analysis showing the distribution of wound sizes according to the different measurement methods.

Descriptive statistics for the three measurements are shown in Table 3. The average values for the ruler method, LiDAR technique, and ImageJ analysis were 112.99 ± 110.07 cm^2^, 73.59 ± 72.97 cm^2^, and 74.29 ± 72.15 cm^2^, respectively, and the difference between the ruler method and ImageJ analysis and LiDAR technique and ImageJ analysis were 39.40 and 0.7, respectively. Pearson correlation analysis revealed that the r values of the ruler and LiDAR techniques compared to those of ImageJ analysis (gold standard method) were 0.990 and 0.995, respectively (Table 4). In the box plot, the measurements of the LiDAR technique were closer to those of the gold standard than those of the conventional ruler method (Figure 3). In the linear regression analysis, the LiDAR technique showed a slope value close to 1 compared to that of the ruler method, which indicates that the LiDAR technique closely matches the gold standard (Figure 4).

**Figure 5:**
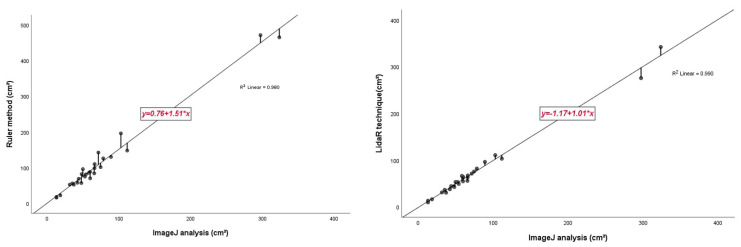
Regression analysis results for measurements using the ruler method, LiDAR, and ImageJ analysis. The regression equation is expressed as: y = a (95% CI) + b (95% CI) × (Passing and Bablok regression). (**Left**) The regression line between the ruler method and Image J analysis measurements. The regression line has a slope of 1.51 and an intercept of 0.76. The correlation coefficient between the two methods is r = 0.980. (**Right**) The regression line between the LiDAR method and Image J analysis measurements. The regression line has a slope of 1.01 and an intercept of 1.17. The correlation coefficient between the two methods is r = 0.990. Note that the correlation coefficient was relatively high for the LiDAR technique and the slope of the linear regression was approximately 1, thus indicating a high degree of accuracy.

**Figure 6:**
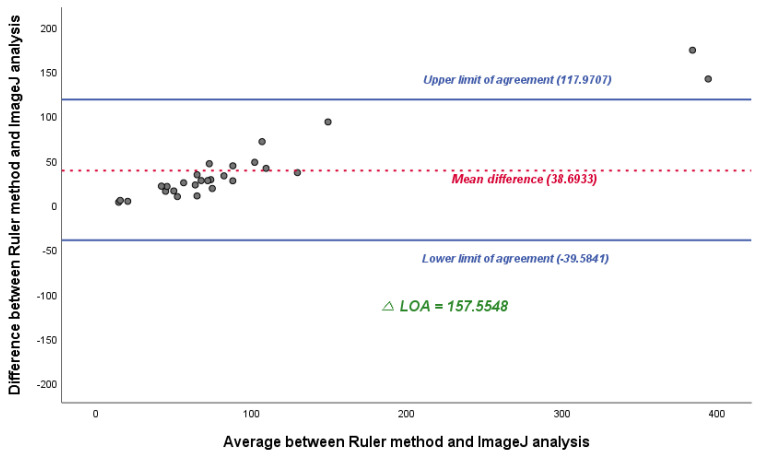

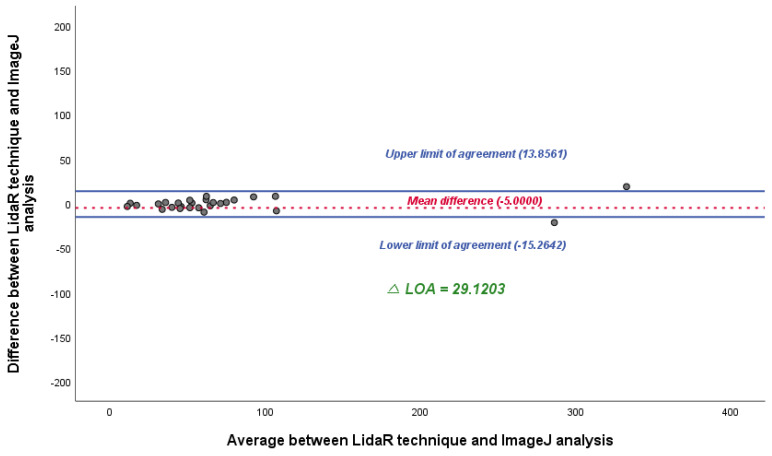
Bland–Altman plot between the ruler method and ImageJ analysis (**top**) and the LiDAR technique and ImageJ analysis (**bottom**). Note that a significant difference of agreement is observed.

Although the Pearson correlation coefficient value was observed to be relatively high for the LiDAR technique (0.995), it did not show a significant difference(Figure 5); therefore, an additional Bland–Altman plot was constructed. Compared with linear regression, we observed a considerable difference and found that the mean difference and △LOA (upper limit of agreement–lower limit of agreement) for the two different measurement methods were 38.6933 and 157.5548, and −5.0000 and 29.1203, respectively. It was noted that the △LOA of the ruler method was approximately five times higher than that of the LiDAR technique, which implies that the LiDAR technique is statistically more accurate than the ruler method (Figure 6).

### 3.2. Representative Patients

**Figure 7:**
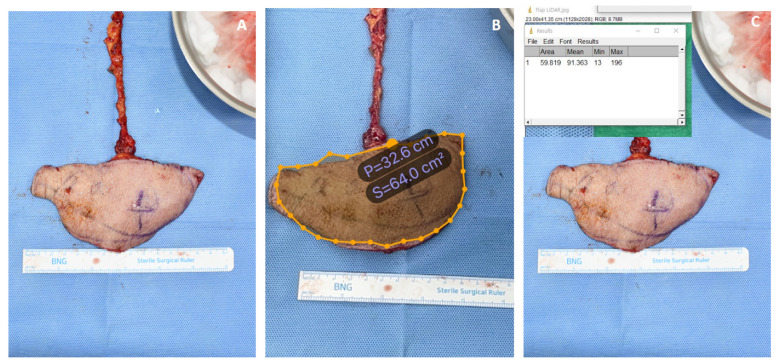
Comparison of the measured wound size for patient #1. (**A**) Conventional Ruler method (**B**) LiDAR technique (**C**) ImageJ analysis.

Patient #1: An 81-year-old male underwent free-flap surgery for a diabetic foot ulcer. A free flap from the right thoracodorsal artery perforator was harvested from each patient. The flap size was assessed using the three aforementioned methods. The flap was oval and flat. The estimated size of the mass measured using the conventional rectangular method was 78.0 cm^2^ (13.0 cm × 6.0 cm). The estimated size of the mass obtained using LiDAR was 64.0 cm^2^. For an accurate comparison, the areas were also calculated using ImageJ software, and the result was 59.8 cm^2^ (Figure 7).

**Figure 8:**
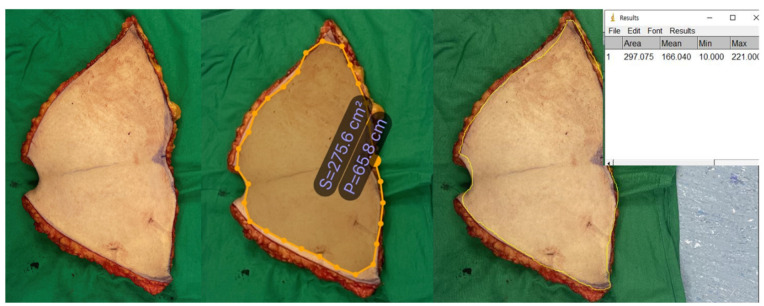
Comparison of the measured wound size for patient #2.

Patient #2: A 40-year-old female underwent an abdominoplasty for rectal diastasis. An excessive skin flap was resected from each patient. The flap size was assessed using the three aforementioned methods. The flap was oval and flat. The estimated size of the mass obtained using the conventional rectangular method was 471.04 cm^2^ (29.44 cm × 16.0 cm). The estimated size of the mass obtained using LiDAR was 285.6 cm^2^. For an accurate comparison, the areas were also calculated using Image J software, and the result was 297.08 cm^2^ (Figure 8).

## 4. Discussion

**Figure 9:**
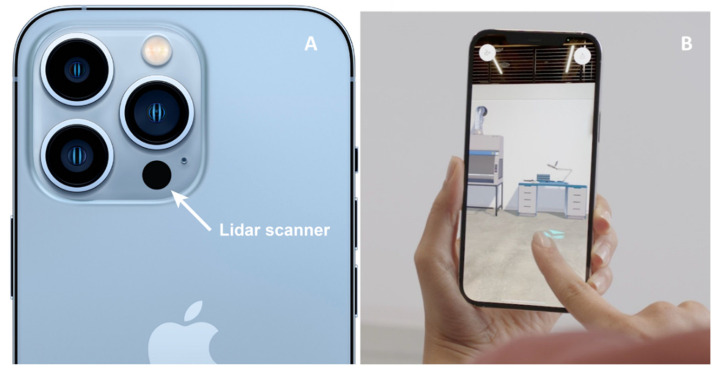
The LiDAR application. (**A**) Apple iPhone 12 Pro camera module with a LiDAR scanner on the bottom. (**B**) Scanning of an in-house object with an iPhone 12 Pro.

Wound measurement plays a central role in the successful management of advanced wounds. The regular assessment of wound size can predict healing and provides information that can guide treatment decisions [1,5]. The current methods used for wound size measurement in clinical settings are centered on simple ruler-based techniques or wound tracing. These approaches are limited by subjective interpretation, significant inter-observer variability, and a high degree of inaccuracy [2]. Wound measurement tools for advanced wound care using various modalities are becoming available in the market. However, their disadvantages include high prices, the need for expensive machines for measurement, and the inconvenience of measurement. Most importantly, although these advanced wound assessment modules are accurate, they require probes for estimation. To overcome these disadvantages, we introduced a LiDAR sensor to assess wound size (Figure 9). LiDAR is a well-established technique commonly used to measure distances by timing the return pulse emitted from a laser transmitter to a laser receiver [6,7,8,9].

The difference between our application and the built-in application on the iPhone is that it allows the calculation of the area in two dimensions rather than a 1-dimensional length. Furthermore, this application can be used immediately after downloading for free from the app store, and the time required for the measurement does not differ from that required to take a picture with a conventional smartphone camera.

For a wound measurement technique to be useful in clinical practice and research, it needs to be time- and cost-efficient, easy to use, and should minimize patient discomfort [10].

In this study, the LiDAR technique application for wound size assessment was developed and validated for wound size measurement in actual patients. This method is advantageous, as it does not require expensive cameras or any specific equipment. It is based on free-of-charge applications that can be downloaded from the app store. Overall, in our study, we demonstrated that the LiDAR technique is more accurate than the conventional ruler method. Furthermore, traditional methods, such as wound tracing on a transparent film, can be uncomfortable for patients [11,12,13]. In contrast, LiDAR-based measurements are non-invasive and can be performed quickly, thereby reducing patient discomfort when assessing wound size. Additionally, this technique reduces the risk of infection, as the LiDAR technique eliminates the risk of wound contamination associated with tracing on transparent film or direct contact with rulers [4]. This is especially critical in wound assessment, as wound infections can lead to severe complications.

From the perspective of reconstructive surgery, the results of this study have various clinical implications. First, the precise estimation of defects results in the minimal harvest of autologous donor tissue. Thus, surgeons can better estimate the amount of tissue needed for grafts or flaps, reducing the risk of underestimation or overestimation and resulting in more efficient surgeries with fewer complications because an overestimated defect size requires an oversized flap elevation, which increases the chance of flap necrosis [14]. Second, owing to the real-time assessment capability of the LiDAR application, surgeons can use the LiDAR technique to accurately measure wounds and defects on the spot, thus helping them determine the most appropriate surgical approach.

However, this study has some limitations. First, owing to the technical limitations of mobile devices, the number of included patients (28) in this study was smaller than the number of patients evaluated (45). Second, wounds smaller than 2.0 × 2.0 cm^2^ were not included in this study because of the technical limitations of the LiDAR technique in smartphone devices. Wounds below this size cannot be measured because the size of the dot on which the application was segmented was larger than the size of the mass. Third, the wound margin was manually identified by the physician, without using an automated method; this could potentially introduce measurement bias and decrease the accuracy of the result. Fourth, the wound was limited to the flap and corresponding defect. This is because a non-flat wound cannot be bound by the LiDAR technique; this limitation should be addressed in future research. A two-dimensional measurement of the surface area that reflects depth should be devised in future studies. Finally, in the application used in this study, users manually assessed the boundary with their own hands, which may have introduced errors.

To overcome these limitations, future studies should focus on the development of computational algorithms for estimating the surface area of wounds with uneven depth, such as pressure ulcers, atypical shaped masses, and concave or convex ulcerative lesions or defects, using the LiDAR technique with mobile devices.

## 5. Conclusions

The LiDAR application reported in this paper offers a simple and accurate method for measuring wound size, particularly for wounds with irregular shapes. It has the potential to improve wound-care management by providing precise measurements and reducing patient discomfort. Moreover, the precise measurement of the wound or defect would result in minimal donor site sacrifice in terms of reconstructive surgery. Future studies should focus on addressing the limitations of the LiDAR technique, particularly with respect to non-flat and smaller wounds, and on developing computational algorithms to estimate the surface area of wounds with uneven depths.

## Figures and Tables

**Table 1:** Comparison of the different wound size assessment methods.

Methods	Advantages	Disadvantages	Examples
Traditional rectangular method	Simplest measurement method No additional device is required	Inaccuracies of up to 44% [1,2,3]	Ruler
LiDAR-based assessment	Easy to use No probe required for calibration	Unknown: no prior studies have investigated this technique	AR Ruler App: tape Measure Cam (not for wound analysis)
ImageJ surface analysis	Most common method used to assess size measurement Easy to use Can measure complex objects	Image files must be transferred to a computer Probe necessary for calibration	Wound size measurement Determination of the photosynthetic portion of a variegated leaf Particle counting in molecular biology

**Table 2:** iPhone 12 Pro Max key specification used in this study and a list of cell phones (and devices) that use LiDAR technology.

Hardware Information
iPhone 12 PRO MAX Key Specifications: Screen: 6.7 inches. Processor: Apple A14 Bionic. Storage: 128, 256, and 512 GB. Camera: Three 12-megapixel rear cameras; 12-megapixel front-facing camera. Weight: 226 g. List of cell phones (and devices) that Apple produces that use LiDAR technology iPhone 14 Pro Max iPhone 14 Pro iPhone 13 Pro Max iPhone 13 Pro iPhone 12 Pro iPhone 12 Pro Max iPad Pro (2020 Version and Later)

**Table 3:** Descriptive statistics and box plot analyses of the actual wound sizes.

	Ruler	LiDAR	ImageJ
N	28	28	28
Average	112.99	73.59	74.29
Standard Deviation (SD)	110.07	72.97	72.15
Range	455.05	332.90	310.56
Minimum	15.99	9.40	12.80
Maximum	471.04	342.30	323.36

**Table 4:** Correlation analysis among the three different measurement methods (bivariate analysis).

		Ruler	LiDAR	ImageJ
**Ruler**	Pearson Correlation	1	0.984 **	0.990 **
	Sig. (2-tailed)		0.000	0.000
	N	28	28	28
**LiDAR**	Pearson Correlation	0.984 **	1	0.995 **
	Sig. (2-tailed)	0.000		0.000
	N	28	28	28
**ImageJ**	Pearson Correlation	0.990 **	0.995 **	1
	Sig. (2-tailed)	0.000	0.000	
	N	28	28	28

** Correlation is significant at the 0.01 level (2-tailed).

## Data Availability

The data presented in this study are available upon request from the corresponding author. The data are not publicly available due to patients’ privacy.

## Supplementary Materials

The following supporting information can be downloaded at: https://www.mdpi.com/article/10.3390/jcm12186042/s1. Supplementary Video S1. Demonstration of the use of the LiDAR application for actual wound size assessment in the operating field.
