# Supplementary material for: Smartphone-Based LiDAR Application for Easy and Accurate Wound Size Measurement
Source: J Clin Med. 2023 Sep 19;12(18):6042. doi: 10.3390/jcm12186042 (PMC10531604; doi:10.3390/jcm12186042)
Supplement: Supplementary file 1 [file jcm-12-06042-s001.zip › Supplementary_material.pdf]

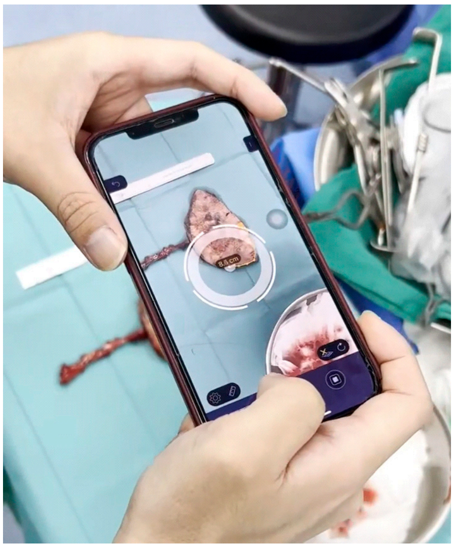

Supplemental video S1. Demonstration of the use of the LiDAR application for actual wound size assessment in the operating field.
